# Analysis of anterior corneal surface shape after replacing orthokeratology lenses carrying a small base curve diameter

**DOI:** 10.3389/fnins.2024.1424394

**Published:** 2024-11-18

**Authors:** Minfeng Chen, Sijun Zhao, Lulu Peng, Yu Rong, Chengwei Zhu, Fan Lu, Xinjie Mao

**Affiliations:** Optometry Clinic Center, Wenzhou Medical University Eye Hospital, Wenzhou, Zhejiang, China

**Keywords:** myopia, orthokeratology, base curve, axial length, decentration distance

## Abstract

**Purpose:**

The study analyzed the changes in corneal surface shape after replacing orthokeratology lenses carrying a small base curve (BC) diameter.

**Methods:**

In this retrospective study, we included ~54 right eyes belonging to 54 myopic children who insisted on wearing an orthokeratology (ortho-k BC 6.0 mm) lens for more than 12 months and then replaced the second ortho-k (BC 6.0 mm or BC 5.0 mm) lens ~12 months. The children were categorized into two groups based on the design of the BC of the replaced ortho-k lens 6.0/5.0 and 6.0/6.0.

**Results:**

The ratio of axial length (AL) elongation in the 6.0/5.0 group was significantly less than in the 6.0/6.0 group (−0.015 ± 0.014 mm/M vs. −0.005 ± 0.012 mm/M, *t* = −2.672, *P* = 0.010). After replacing the BC 5.0 ortho-k lens, the optical zone (8.19 ± 2.60 mm^2^ vs. 9.64 ± 1.57 mm^2^, *t* = −2.345, *p* = 0.023), reverse zone (31.64 ± 5.80 mm^2^ vs. 34.86 ± 4.61 mm^2^, *t* = −2.169, *p* = 0.035), and treatment zone (17.16 ± 3.94 mm^2^ vs. 22.96 ± 2.59 mm^2^, *t* = −6.049, *p* < 0.001) were all smaller than those in the 6.0/6.0 group. In the 6.0/5.0 group, the optical zone (wearing more than 1 month as first: 11.16 ± 2.12 mm^2^, the last inspection before replacing lens as before: 10.87 ± 1.90 mm^2^), reverse curve zone (first: 22.03 ± 3.11 mm^2^, before: 26.24 ± 5.06 mm^2^), and treatment zone (first: 35.97 ± 5.54 mm^2^, before: 37.11 ± 6.04 mm^2^) were all greater than these after replacing ortho-k lens (all *P* < 0.001).

**Conclusion:**

Replacing an ortho-k lens with a smaller BC resulted in a larger decrease in the mean central corneal zone and a substantial increase in the paracentral corneal zone. In addition, the ortho-k lens with a smaller BC does not increase the decentration distance and contributes to effective myopia control.

## Introduction

Due to the lack of effective interventions against progressive myopia, the incidence of myopia is expected to account for ~50% of the global population by 2050, including 938 million cases progressing to high myopia (Holden et al., 2016). High myopia increases the risk of retinal detachment, glaucoma, macular degeneration, and other retinal diseases and thus represents a global public health challenge (Ikuno, 2017; Chen et al., 2012; Marcus et al., 2011). Orthokeratology (ortho-k) lenses have been used as a clinical non-surgical method for the temporary correction of myopia and to delay the progression of myopia in children. Ortho-k lenses are a safe, effective, and reversible mode of intervention againsth myopia (Li et al., 2016; Chen et al., 2023; Lin et al., 2023; Hiraoka et al., 2015; Chen J. et al., 2018; Na and Yoo, 2018; Huang et al., 2023).

Nevertheless, the mechanism of myopia control with ortho-k lenses has not been fully understood. It is generally agreed that an ortho-k lens can reshape the anterior corneal surface. The unique anti-geometric design of the ortho-k lens can flatten the central corneal surface and steepen the paracentral corneal surface. It therefore transforms the hyperopia into myopic defocus imaging in the peripheral retina to control myopia (Chen et al., 2023; Lin et al., 2023; Hiraoka et al., 2015; Chen J. et al., 2018; Na and Yoo, 2018; Huang et al., 2023; Ding et al., 2023).

However, the effect of ortho-k lenses on myopia control varies among children with myopia. Studies suggest that reducing the base curve (BC) diameter of an ortho-k lens can control myopia effectively (Marcotte-Collard et al., 2018; Gifford et al., 2020; Guo et al., 2021; Zhang et al., 2022; Kang and Swarbrick, 2016; Chen et al., 2020). Hu et al. (2019) reported that children with myopia carrying a smaller reshaping zone after wearing an ortho-k lens showed slower axial length (AL) elongation. Furthermore, the ortho-k lens with a smaller BC diameter resulted in a small reshaping zone (Gifford et al., 2020).

Corneal topography, using a Medmont Topographer, has been used to effectively analyse the reshaping zone of the anterior corneal surface after wearing an ortho-k lens (Gifford et al., 2020; Guo et al., 2021; Zhang et al., 2022; Kang and Swarbrick, 2016; Chen et al., 2020; Hu et al., 2019; Chen Z. et al., 2018; Wang and Yang, 2016; Li et al., 2022, 2017; Chen et al., 2017; Carracedo et al., 2019). Thus, the optical zone and the reverse zone have been analyzed via corneal tangential difference topography, and the treatment zone has been analyzed using axial difference topography. Discontinuation of the ortho-k lens led to a slow recovery of the shape of the anterior corneal surface to the original shape, while the flat radial refraction was reduced. Although replacing the second ortho-k lens might be worth considering, it is difficult to prevent children from wearing the lens for a prolonged duration to ensure that the anterior corneal surface shape is restored to its original shape. Furthermore, it is unclear whether the ortho-k lenses with a small BC diameter are effective even after replacement.

The present study aimed to investigate the difference in corneal topography between patients with replaced ortho-k lenses carrying a small BC diameter and those with replaced orthokeratology lenses carrying a normal BC diameter. The corneal surface shape after replacing the ortho-k lenses without discontinuation was also analyzed.

## Methods

### Subjects

A total of 54 children with myopia who insisted on wearing BC 6.0-mm ortho-k lens for ~12 months and replaced the second ortho-k (BC 6.0 mm or BC 5.0 mm) lens ~12 months were included in this study from January 2021 to January 2024 at the Wenzhou Medical University Eye Hospital. Based on the BC design of the replaced ortho-k lens, base curve (BC) 5.0 (BC diameter, 5.0 mm) and 6.0 (BC diameter, 6.0 mm), the subjects were divided into two groups in this study (6.0/5.0 group and 6.0/6.0 group). The inclusion criteria were as follows: (1) age: 8–14 years; (2) a diagnosis of myopia, best-corrected visual acuity was equal to or better than 0.1 (LogMAR) in each eye, spherical equivalent refraction (SER) ranging from −1.00 D to −6.00 D, and astigmatism ranging from −1.50 D to 0.00 D; (3) no history of using atropine for myopia, including this entire follow-up period; (4) no history of other eye diseases (such as trauma, strabismus, amblyopia, and binocular vision anomalies); and (5) no history of surgery and serious systemic diseases. This article follows the principles of the Declaration of Helsinki and was approved by the Wenzhou Medical University Eye Hospital's Ethics Committee (2023-107-K-86).

### Instrumentation

Both the BC 6.0-mm and BC 5.0-mm design ortho-k lenses analyzed in this study were three-zoned reverse-geometry lenses (CRT, USA). The ortho-k lens parameters are shown in Table 1. All subjects accepted a complete eye examination at baseline time, including a slit-lamp examination, refraction, best-corrected visual acuity, AL (IOL Master; Zeiss), corneal topography (Medmont International Pty. Ltd.), and intraocular pressure (NT-2000; NIDEK). All children were treated by doctors who had worked in the field of ortho-k lenses at the hospital for more than 10 years. Within 1 h of removing the ortho-k lenses, the corneal topography was obtained by the specialized technician through the Medmont E-300. According to the best-fitting evaluation based on corneal fluorescein pattern analysis, the doctor ordered the most suitable lens for the subject. All subjects wore the first ortho-k lens with a base curve of 6.0 mm for more than 12 months (6.0/5.0 group: *n* = 32, 12.91 ± 1.49 months and 6.0/6.0 group: *n* = 22, 13.45 ± 1.97 months). When the ortho-k lenses needed to be replaced, they were replaced with a base curve of 5.0 mm in some children (12.94 ± 2.20 months) and with a base curve of 6.0 mm in others (13.05 ± 1.99 months).

**Table 1:** Contact lens parameters used in this study.

**Parameter**	**6.0-mm OZ lens design**	**5.0-mm OZ lens design**
Manufacturer	Paragon vision sciences (Gilbert, AZ)	Paragon vision sciences (Gilbert, AZ)
Brand	Paragon CRT	Paragon CRT
Overall diameter	10.50	10.50
Optic zone diameter	6.00	5.00
Reverse curve (RZD) width	1.00	1.00
Landing curve (LZA) width	1.00	1.50
Power fitted (D)	+0.50	+0.50
Back optic zone radius (mm)	7.90–8.90	7.90–8.90

### Measurement of the shaping zone and decentration distance

**Figure 1:**
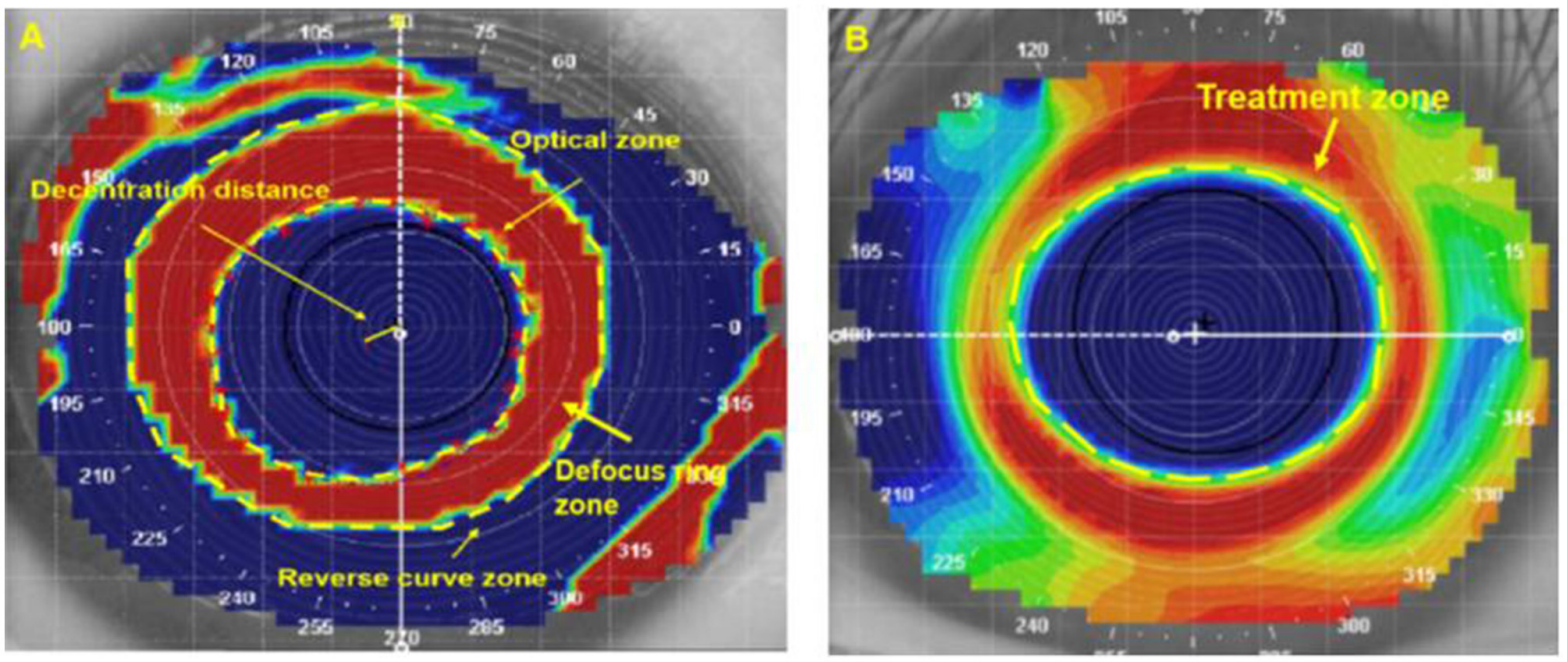
Reshaping zone, decentration distance in the tangential difference topography, and the treatment zone in the axial difference topography. **(A)** The optical zone was the central blue area, the reverse zone was the central red and blue area, the defocus ring zone was the central red area, and the decentration distance measured from the corneal vertex to the center to the optical area; **(B)** the treatment zone was the central blue area.

In the 6.0/5.0 group, the tangential difference topography was used at first (after wearing the first ortho-k lens for more than 1 month), before (the last inspection before replacing the ortho-k lens), and after replacing the ortho-k lens more than 1 month later. The 6.0/6.0 group was analyzed via tangential difference topography at first and after the ortho-k replacement. After spanning from the corneal vertex to the point where the keratometry values changed within 1.00 D to present two colors (red and blue) in the refractive power parameters, the shaping zone (optical zone, reverse zone, and defocus ring zone) in tangential difference topography and treatment zone in axial difference topography were measured, as shown in Figures 1A, B. Tangential difference topography was processed by MATLAB; then, 16 points were selected clockwise in the red–blue transition area to describe the edge of the optical area. The center of the optical zone was positioned by MATLAB. The corneal vertex was located by the Medmont E-300. The decentration distance was defined from the corneal vertex to the center to the optical area. The area of the shaping zone (optical zone, reverse zone, and defocus ring zone) and decentration distance was measured through the ImageJ software (Chen et al., 2020; Hu et al., 2019; Chen Z. et al., 2018; Wang and Yang, 2016; Li et al., 2022, 2017; Chen et al., 2017; Carracedo et al., 2019; Li et al., 2018; Yang et al., 2005).

### Changes in corneal refraction

**Figure 2:**
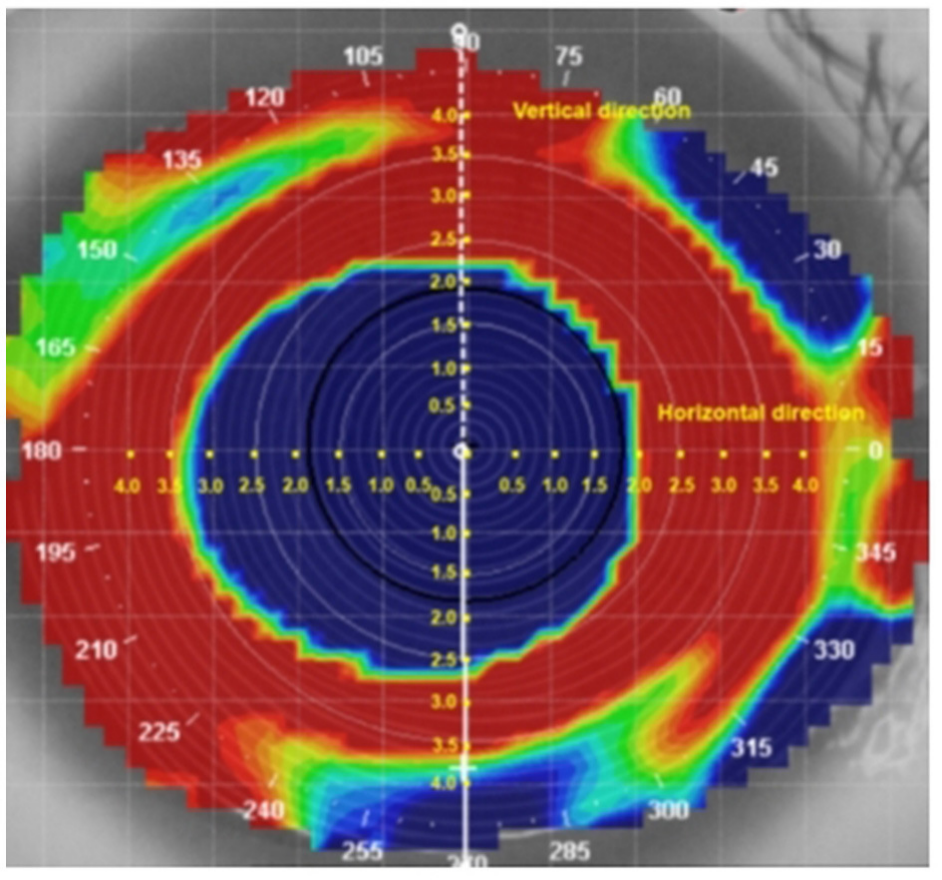
Measurement of the change in refraction along the horizontal and vertical meridians in the axial difference topography.

The changes in refraction along the horizontal and vertical meridians from the axial difference topography were measured at intervals of 0.5 mm (Guo et al., 2022; Liu et al., 2023; Kang et al., 2021; Wang and Yang, 2019). According to the data presented in Figure 2, the analysis was performed on ~33 points of refraction changes in both the horizontal and vertical meridians of the corneal topography.

### Statistical analysis

The data were processed using SPSS 24.0 for the Windows statistical software. The measurement data were expressed by *X* ± *s*. Data were first tested for normality using the sample Shapiro–Wilk test. The independent sample *t*-test was used to analyze the differences in group parameters. The paired sample *t*-test was used to analyze the changes in parameters before and after replacing the ortho-k lens in the 6.0/6.0 group. ANOVA was used to analyze the changes in parameters before and after replacing the ortho-k lens in the 6.0/5.0 group. A *P*-value of <0.05 was considered statistically significant. SPSS 25 version was used for statistical analyses (SPSS, IBM, Chicago, IL, USA).

## Results

### Basic information

**Figure 3:**
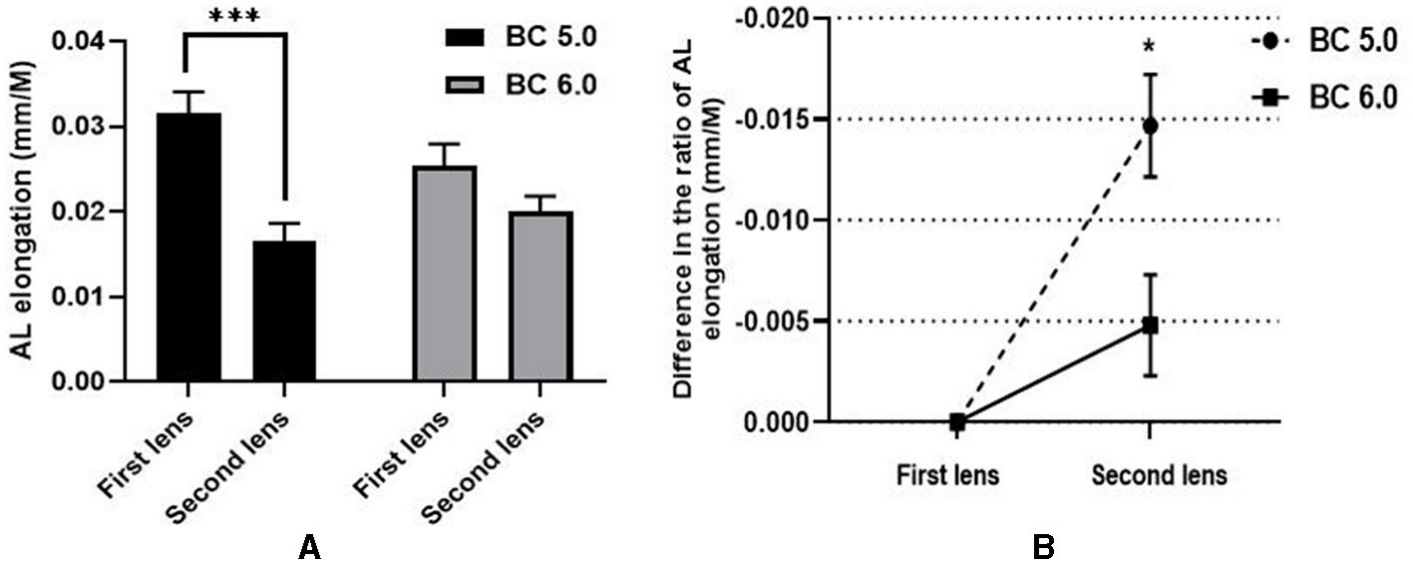
Comparison of the AL elongation between two groups [**(A)** comparison of the difference in AL elongation between the first lens and second lens in two groups; **(B)** comparison of the difference in the ratio of AL elongation between wearing the first ortho-K lens and wearing the second ortho-k lens in two groups]. *P < 0.05, ***P < 0.001.

Approximately 54 right eyes from 54 myopic children (average age: 9.19 ± 1.20 years) were included. For all subjects, the average SER and AL were −2.70 ± 1.20 D and 24.67 ± 0.79 mm, respectively. The 6.0/5.0 group included 32 eyes, and the 6.0/6.0 group had 22 eyes. There were no statistical differences in basic parameters between the two groups. During ortho-k lens treatment, the AL elongation ratio in the second period was significantly lower than in the first period when wearing the ortho-k lens, as illustrated in Table 2. In the 6.0/5.0 group, the ratio of AL elongation with first lens (0.032 ± 0.015 mm/M) was statistically higher than that with second lens (0.017 ± 0.012 mm/M, *t* = 5.784, *P* < 0.001), but there were no significant differences in the AL elongation between the first lens (0.025 ± 0.011 mm/M) and the second lens in the 6.0/6.0 group (0.020 ± 0.008 mm/M, *t* = 1.921; *P* = 0.068). The ratio of AL elongation in the 6.0/5.0 group was significantly lower than that in the 6.0/6.0 group (−0.015 ± 0.014 mm/M vs. −0.005 ± 0.012 mm/M, *t* = −2.672, *P* = 0.010), as shown in Figure 3.

**Table 2:** Baseline parameters of the two groups.

**Groups**	**6.0/5.0 (*n* = 32)**	**6.0/6.0 (*n* = 22)**	***t***	***P***
Gender (male/female)	13/19	13/9		
Age (year)	9.03 ± 1.23	9.41 ± 1.14	−1.141	0.259
SER (D)	−2.63 ± 1.32	−2.80 ± 1.04	0.505	0.616
SE (D)	−2.47 ± 1.23	−2.57 ± 0.98	0.318	0.752
Astigmatism (D)	−0.31 ± 0.45	−0.45 ± 0.41	1.152	0.255
AL (mm)	24.60 ± 0.86	24.77 ± 0.67	−0.781	0.438
HVID (mm)	12.27 ± 0.33	12.12 ± 0.43	1.485	0.143
FK (D)	42.80 ± 1.60	42.80 ± 1.19	0.002	0.999
SK (D)	43.93 ± 1.79	44.01 ± 1.24	−0.197	0.845
Fe	0.64 ± 0.07	0.61 ± 0.11	1.449	0.154
PD (mm)	4.34 ± 0.82	4.40 ± 1.02	−0.252	0.802

SER, spherical equivalent refraction; SE, spherical refraction; AL, axial length; HVID, horizontal visible iris diameter; FK, flat radial refraction; SK, steep radial refraction; Fe, flat radial eccentricity; PD, pupil diameter.

### Shaping zone differences between the 6.0/5.0 and 6.0/6.0 group after wearing an ortho-k lens

No significant differences were found in the parameters between two the groups initially. After replacing the BC 5.0-mm ortho-k lens, the optical zone (8.19 ± 2.60 mm^2^ vs. 9.64 ± 1.57 mm^2^, *t* = −2.345, *p* = 0.023), reverse zone (31.64 ± 5.80 mm^2^, 34.86 ± 4.61 mm^2^, *t* = −2.169, *p* = 0.035), and treatment zone (17.16 ± 3.94 mm^2^, 22.96 ± 2.59 mm^2^, *t* = −6.049, *p* < 0.001) were all smaller than in the 6.0/6.0 group. However, no statistically significant differences in the decentration distance and defocus ring zone were found between the two groups (Table 3).

**Table 3:** Comparison of the differences in corneal reshaping between 6.0/5.0 and 6.0/6.0 groups.

	**Groups**	**6.0/5.0 (*n* = 32)**	**6.0/6.0 (*n* = 22)**	***t***	***P***
First ortho-k lens	Optical zone (mm^2^)	11.16 ± 2.12	10.34 ± 1.98	1.443	0.155
	Reverse curve zone (mm^2^)	35.97 ± 5.54	35.91 ± 4.78	0.604	0.548
	Defocus ring zone (mm^2^)	24.81 ± 4.73	24.76 ± 3.73	0.044	0.964
	Decentration distance (mm)	0.46 ± 0.21	0.47 ± 0.17	−0.257	0.798
	Treatment zone (mm^2^)	22.03 ± 3.11	23.03 ± 2.93	−1.189	0.240
Replacing ortho-k lens	Optical zone (mm^2^)	8.19 ± 2.60	9.64 ± 1.57	−2.345	0.023
	Reverse curve zone (mm^2^)	31.64 ± 5.80	34.86 ± 4.61	−2.169	0.035
	Defocus ring zone (mm^2^)	23.57 ± 3.70	25.22 ± 4.00	−1.663	0.102
	Decentration distance (mm)	0.44 ± 0.21	0.46 ± 0.15	−0.455	0.651
	Treatment zone (mm^2^)	17.16 ± 3.94	22.96 ± 2.59	−6.049	<0.001

### Shaping zone before and after replacing the ortho-k lens

**Figure 4:**
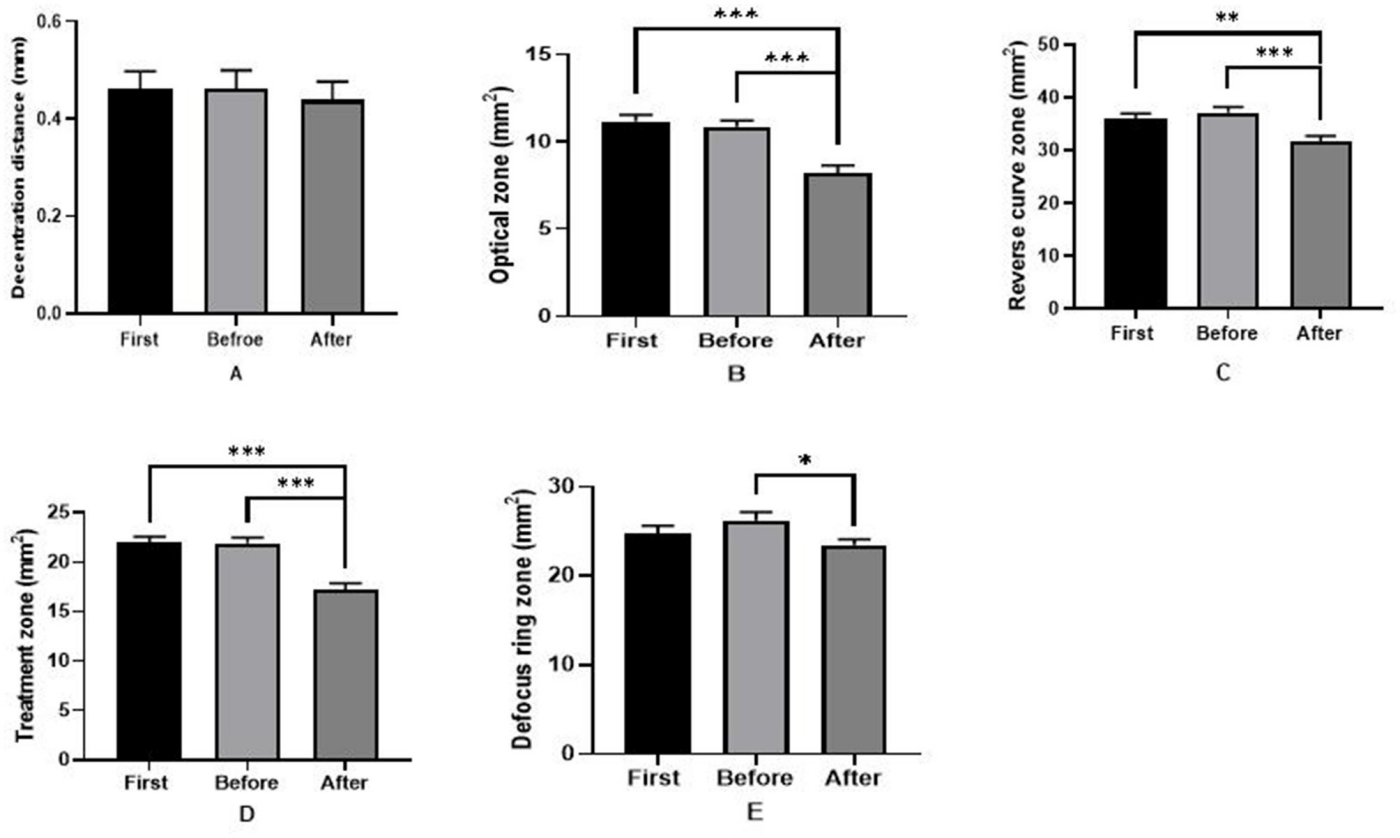
Reshaping zone differences in the 6.0/5.0 group before and after replacing the ortho-k lens. **(A)** Decentration distance difference; **(B)** optical zone difference; **(C)** reverse curve zone difference; **(D)** treatment zone difference; **(E)** defocus ring zone difference (first: after wearing the first ortho-k lens for more than 1 month, before: the last inspection before replacing the ortho-k lens, after: replacing the ortho-k lens more than 1 month later). *P < 0.05, **P < 0.01, ***P < 0.001.

**Figure 5:**
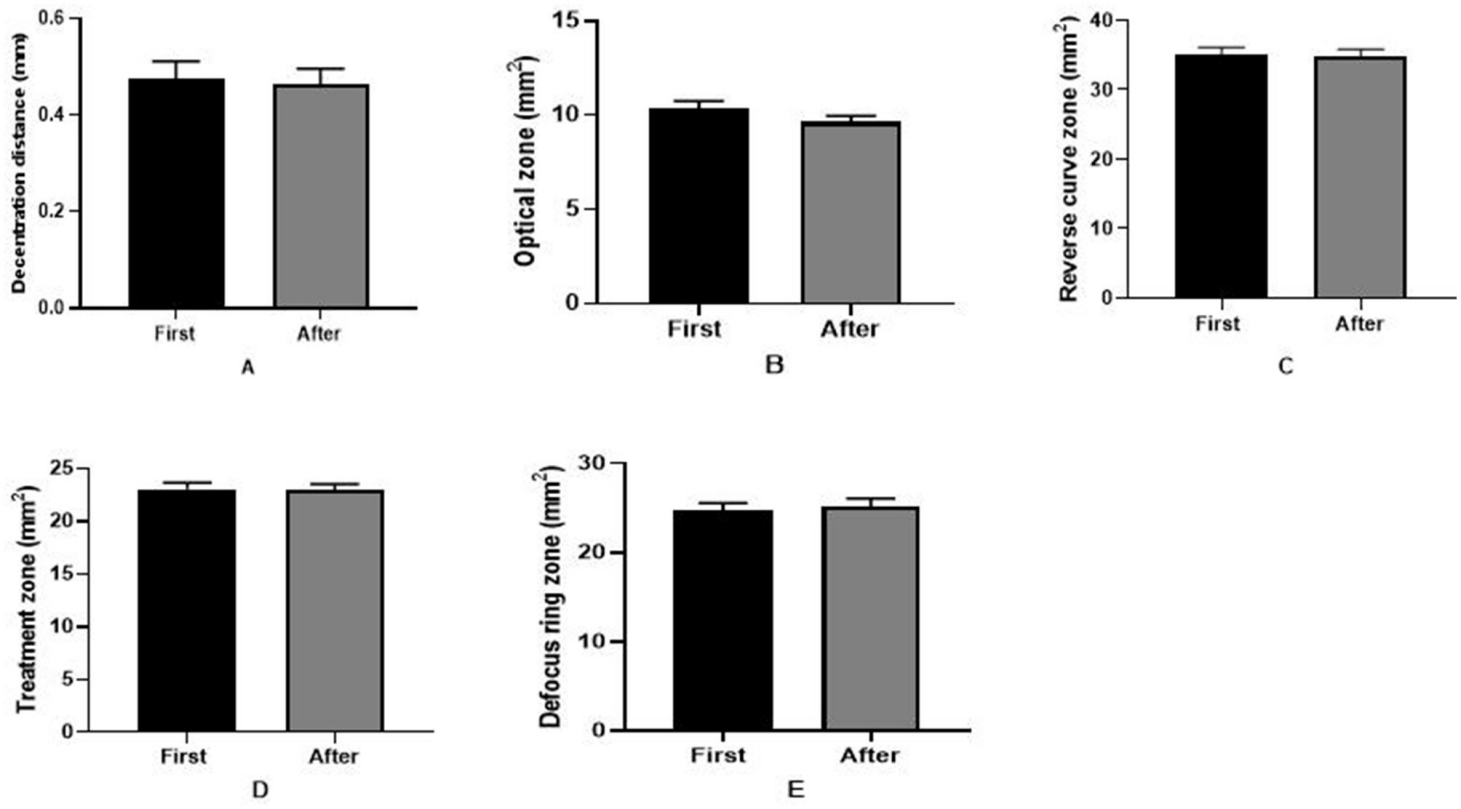
Reshaping zone differences in the 6.0/6.0 group after and before replacing the ortho-k lens. **(A)** Decentration distance difference; **(B)** optical zone difference; **(C)** reverse curve zone difference; **(D)** treatment zone difference; **(E)** defocus ring zone difference (first: after wearing the first ortho-k lens for more than 1 month, after: replacing the ortho-k lens more than 1 month later).

As shown in Figure 4, in the 6.0/5.0 group, the sizes of the optical zone (first: 11.16 ± 2.12 mm^2^, before: 10.87 ± 1.90 mm^2^), reverse curve zone (first: 22.03 ± 3.11 mm^2^, before: 26.24 ± 5.06 mm^2^), and treatment zone (first: 35.97 ± 5.54 mm^2^, before: 37.11 ± 6.04 mm^2^) were all greater initially than after replacing the ortho-k lens (all *P* < 0.001). However, in the 6.0/6.0 group, no significant differences were detected in the shaping zone before and after the ortho-k lens was replaced (Figure 5). Furthermore, the decentration distance was almost stable during the whole period in both groups.

### Refraction changes of the anterior corneal surface before and after replacing the ortho-k lens in the 6.0/5.0 and 6.0/6.0 groups

**Figure 6:**
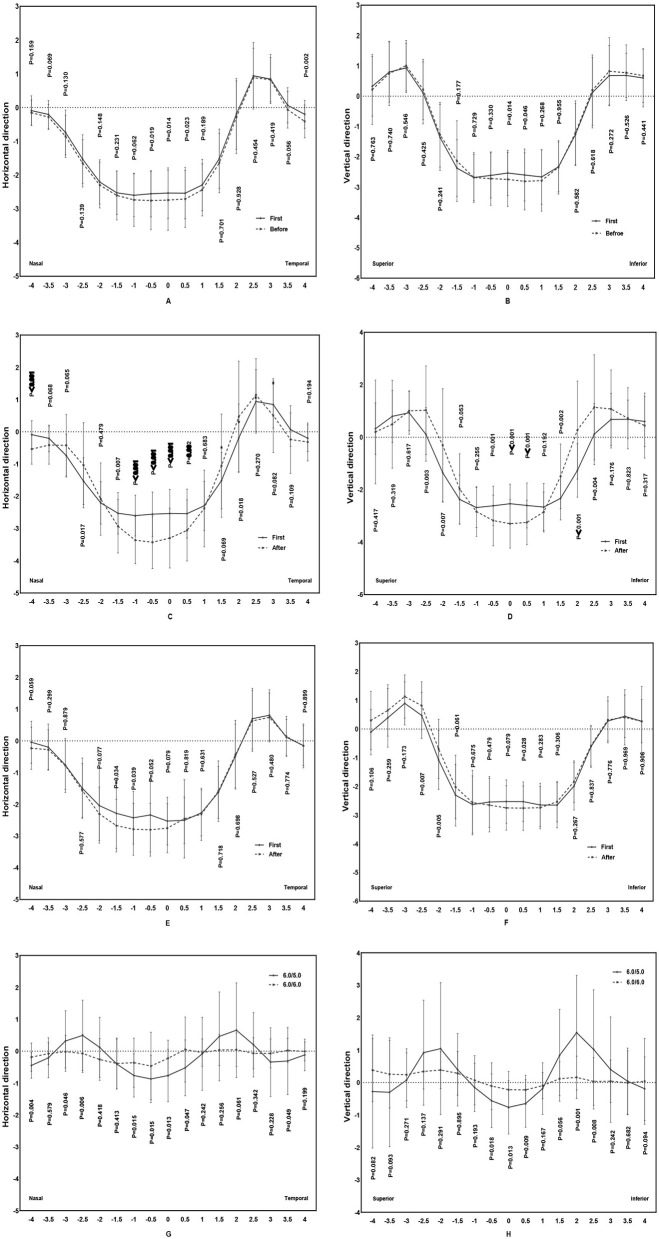
Comparison of the difference in anterior corneal surface refraction in the 6.0/5.0 group and 6.0/6.0 group [**(A, B)** comparison of the difference in anterior corneal surface refraction between first and last ortho-k lenses before replacing in the 6.0/5.0 group, **(C, D)** comparison of the difference in anterior corneal surface refraction between the first and last ortho-k lens after replacing in the 6.0/5.0 group, **(E, F)** comparison of the difference in anterior corneal surface refraction between the first and last ortho-k lens after replacing in the 6.0/6.0 group, and **(G, H)** comparison of the difference in anterior corneal surface refraction change before and after replacing between the 6.0/5.0 and 6.0/6.0 groups].

As shown in Figure 6, the changes in refraction remained almost stable both in the horizontal and vertical directions between the first lens and before replacement in the 6.0/5.0 group. However, the refraction changed markedly after replacing the ortho-k lens in the 6.0/5.0 group, with a larger decrease in the mean central corneal zone and a greater increase in the paracentral corneal zone. However, the corneal refraction in the 6.0/6.0 group changed minimally. Compared with the differences in anterior corneal surface refraction in the 6.0/6.0 group, the corneal refraction changed remarkably in the 6.0/5.0 group both in the central and paracentral zones.

## Discussion

The present study analyzed the reshaped corneal surface in children with myopia undergoing BC 5.0 ortho-k lens replacement and wearing the BC 6.0 ortho-k lens for more than 12 months. Compared with the 6.0/6.0 ortho-k lens replacement group, the 6.0/5.0 group exhibited a larger decrease in the mean central corneal zone and a greater increase in the paracentral corneal zone.

Several factors affect myopia control during ortho-k lens treatment. Guo et al.'s (2021) study reported that the design of the ortho-k lens by reducing the base curve diameter effectively controlled myopia. In this study, after replacing the ortho-k lens, the AL elongation ratio was effectively reduced in both the 6.0/5.0 and 6.0/6.0 groups. Furthermore, the change in AL elongation ratio from the first to the second lens in the 6.0/5.0 group was significantly less than in the 6.0/6.0 group. Thus, replacing the BC 5.0 ortho-k lens enhanced myopia control compared with BC 6.0 ortho-k lens replacement, which was similar to the findings of Guo et al. (2021).

In the 6.0/5.0 group, replacing with the ortho-k lens carrying a smaller base curve reduced the size of the optical zone, reverse curve zone, and treatment zone. However, in the 6.0/6.0 group, the optical zone, reverse curve zone, and treatment zone, which were formed in the second ortho-k lens, were similar to those in the first ortho-k lens. Replacing the lens with a similar base curve design did not change the reshaping zone effectively. The smaller optical zone in the ortho-k lens with a smaller base curve was also reported in Gifford's study (Gifford et al., 2020). The treatment zone in axial difference corneal topography revealed the smaller reshaping zone clearly in the ortho-k lens with a small base curve.

The decentration distance and the defocus ring zone were similar in the second or first ortho-k lenses in both the 6.0/5.0 and 6.0/6.0 groups. The decentration distance in the 6.0/5.0 group was 0.46 ± 0.21 mm in the first lens and 0.44 ± 0.21 mm in the second lens and was smaller than in previous studies (0.64 ± 0.38 mm, 0.51 ± 0.23 mm, and 0.73 ± 0.25 mm; Chen et al., 2020; Wang and Yang, 2019; Gu et al., 2019). Thus, replacing the ortho-k lens carrying the small base curve did not increase the decentration distance and ensured a similar defocus ring zone area. It suggests that the smaller base curve design contributed to the larger width of the landing curve.

Zhong et al. (2014) reported that substantial changes in anterior corneal surface refraction led to effective myopia control after wearing an ortho-k lens. Liu et al. (2023) found that wearing a base curve aspheric (BCA) ortho-k lens resulted in a greater aspheric treatment zone than a base curve spherical (BCS) ortho-k lens. In the 6.0/5.0 group, the changes in anterior corneal surface refraction of the first ortho-k lens were similar to the final changes in anterior corneal surface refraction before replacing the lens. Thus, the changes in anterior corneal surface refraction ensured stability during the first ortho-k lens treatment. However, the changes in anterior corneal surface refraction after replacing the ortho-k lens differed significantly from those associated with the first ortho-k lens. Lens replacement decreased the central zone and increased the paracentral corneal region. The zero point was reached quicker than in the first ortho-k lens. Nonetheless, in the 6.0/6.0 group, the change in anterior corneal surface refraction after replacing the ortho-k lens was insignificant and was similar to the change in the BCA ortho-k lens in Liu's study, which induced a more myopic relative peripheral refraction, contributing to myopia control (Liu et al., 2023).

It has been argued that the differences in maximum corneal refraction within the central 4-mm diameter were negatively correlated with axial elongation in the ortho-k lens (Hiraoka et al., 2015). Hu et al. (2019) found that the total corneal refractive power shift within a 7.2-mm area was a crucial determinant of efficacy after ortho-k lens treatment in children. Chen et al. (2022) demonstrated that increased hyperopic refraction decreased AL elongation. Without stop wearing the ortho-k lens, after replacing the BC 6.0 ortho-k lens, the decentration distance, optical zone, reverse curve zone, and treatment zone all remained stable. However, after replacing the BC 5.0 ortho-k lens, only the decentration distance remained stable, and the optical zone, reverse curve zone, and treatment zone reduced significantly. After replacing the BC 5.0 ortho-k lens, the larger decrease in the mean central corneal zone and the greater increase in the paracentral corneal zone might contribute to the reshaping effect in the corneal surface zone, which resulted in greater hyperopic central refraction with a higher paracentral corneal zone. More areal summed corneal power shift could be found after replacing the BC 5.0 ortho-k lens than the 6.0/6.0 group, which could take better effect on myopia control (Hu et al., 2019).

This is the first study to investigate the effects of the ortho-k lens on corneal topography and refraction after replacing the lens with different a base curve diameter. During the observation period, the uncorrected visual acuity of all subjects after removing the lens was 0.6 logMAR or above. No obvious abnormalities including unclear vision were observed. Neither lens design resulted in any severe adverse events. After replacing the BC 5.0 lens, there were no reported clinically significant adverse effects, except for more initial complaints of halos and slightly worse of visual acuity, which was well-accepted by the subjects in the end. Despite these advantages, the study limitation was that it was difficult to make children stop wearing the ortho-k lens for a long time. Therefore, this study only analyzed ortho-k lens replacement without discontinuation.

## Conclusion

In conclusion, replacing the ortho-k lens carrying a smaller base curve results in a larger decrease in the mean central corneal zone and a greater increase in the paracentral corneal zone. Based on this approach, an ortho-k lens with a smaller base curve does not increase the decentration distance and thereby contributes to effective myopia control.

## Data Availability

The raw data supporting the conclusions of this article will be made available by the authors, without undue reservation.
